# MiR‐486 promotes proliferation and suppresses apoptosis in myeloid cells by targeting Cebpa in vitro

**DOI:** 10.1002/cam4.1694

**Published:** 2018-08-02

**Authors:** Jingwei Jiang, Qingmin Gao, Yiwei Gong, Lizhen Huang, Hao Lin, Xinli Zhou, Xiaohua Liang, Weijian Guo

**Affiliations:** ^1^ Department of Medical Oncology Fudan University Shanghai Cancer Center Shanghai China; ^2^ Department of Oncology Huashan Hospital Fudan University Shanghai China; ^3^ School of Bioscience and Bioengineering South China University of Technology Guangzhou China; ^4^ Department of Oncology Huashan Hospital North Fudan University Shanghai China

**Keywords:** *Cebpa*, microrna, myeloid cells, myeloid‐derived suppressor cells, tumor immune evasions

## Abstract

The monocytic MDSC (M‐MDSC) is one of the major types of MDSCs, which play important roles in suppression of antitumor immunity. However, the mechanisms underlying how M‐MDSCs so heavily accumulate in patients with cancer are still poorly understood. The purpose of this study was to identify miRNAs that regulate the proliferation and differentiation of M‐MDSCs. Microarray analysis was performed to identify differentially expressed miRNAs between tumor‐induced M‐MDSCs (TM‐MDSCs) and their counterparts from tumor‐free mice. The miRNAs and their target genes that regulate the proliferation and differentiation of myeloid cells were predicted by bioinformatics analysis and validated by RT‐qPCR. Luciferase reporter assays were used to analyze the relationships between miRNAs and target genes. Overexpression of candidate miRNAs and target genes in myeloid cells was conducted to verify their functions in cell proliferation, differentiation, and apoptosis. Our data showed that miR‐486 was overexpressed in TM‐MDSCs. *Cebpa* was predicted to be one of the target genes of miR‐486 that regulates the proliferation of myeloid cells. Expression of *Cebpa* was inversely correlated with miR‐486 in TM‐MDSCs, and we found that overexpression of miR‐486 suppressed the expression of *Cebpa* in both 293T cells determined by luciferase reporter assays and in myeloid cells determined by RT‐qPCR. Overexpression of miR‐486 promoted proliferation and suppressed apoptosis in myeloid cells, as opposed to overexpression of *Cebpa,* which promoted the opposing phenotype. Overexpression of either miR‐486 or *Cebpa* inhibited differentiation of myeloid cells. This study indicates that miR‐486 promotes proliferation and suppresses apoptosis in myeloid cells by targeting *Cebpa* in vitro, suggesting that miR‐486 and *Cebpa* might be involved in the expansion of TM‐MDSCs in tumor‐bearing mice.

## 1 BACKGROUND

Tumor‐induced myeloid‐derived suppressor cells (MDSCs) are involved in tumor angiogenesis and evasion of host immunity through T cell suppression. It has been reported that the number of MDSCs in the peripheral blood correlates with prognosis and tumor burden. The phenotype of MDSCs in mice is Gr1^+^CD11b^+^.^1^ Murine MDSCs can be divided into two subsets based on the expression of Ly6C and Ly6G. Monocytic MDSCs (M‐MDSCs), whose phenotype is CD11b^+^Ly6G^−^Ly6C^hi/+^, have a monocyte‐like morphology and express high levels of inducible nitric oxide synthase (iNOS) and arginase 1 (Arg1).^2^, ^3^ Granulocytic MDSCs (G‐MDSCs), whose phenotype is CD11b^+^Ly6G^+^Ly6C^low/+^, not only have a granulocyte‐like morphology, preferentially producing reactive oxygen species (ROS) and expressing Arg1, but also possess a polymorphonuclear (PMN) morphology, explaining why G‐MDSCs are also abbreviated to PMN‐MDSCs.^2^, ^3^ Although G‐MDSCs account for the majority of MDSCs, M‐MDSCs have been found to be no less immunosuppressive than G‐MDSCs on a per cell basis.^2^, ^4^, ^5^ A third subset of MDSCs, namely, more immature, or early, MDSCs (eMDSCs) was recently defined, and the eMDSC phenotype is Lin^−^ (CD3/14/15/19/56) HLA‐DR^−^ CD33^+^ in humans and has yet to be determined in mouse.^6^ Substantial evidence from preclinical and clinical studies has revealed that the expansion of MDSCs in the tumor microenvironment is mediated by a combination of factors, including cytokines and growth factors.^7^, ^8^, ^9^ Although micro ribonucleic acids (miRNAs), which are small noncoding RNAs that posttranscriptionally regulate the production of proteins, account for only 1%‐2% of all genes in mammals, each miRNA is predicted to regulate hundreds of targets. Therefore, the majority of protein coding genes are considered to be under the regulation of miRNAs. Accumulative evidence has shown that miRNAs regulate many cell processes, such as proliferation, differentiation and survival.^10^ Moreover, it was reported that miRNAs also modulate hematopoietic lineage differentiation,^11^ a typical example of which includes myeloid‐specific miR‐223 inhibition of progenitor proliferation, granulocyte differentiation and activation.^12^ There have been several studies validating the hypothesis that MDSCs originating from myeloid cells might also be regulated by miRNAs.^13^, ^14^, ^15^, ^16^ Studies have shown that both miR‐17‐5p and miR‐20a decrease the suppressive potential of G‐MDSCs, while having no effect on M‐MDSCs.^16^ IL‐6‐stimulated CD11b^+^CD14^+^ HLA‐DR^−^ M‐MDSCs are associated with progression and poor prognosis in squamous cell carcinoma of the esophagus.^17^ It was recently reported that ipilimumab treatment decreases M‐MDSCs and increases CD8 effector memory T cells in long‐term survivors with advanced melanoma, indicating that the frequency of M‐MDSCs can predict treatment outcome.^18^ As M‐MDSCs possess special biological characteristics from G‐MDSCs, the miRNA expression profile of tumor‐induced M‐MDSCs (TM‐MDSCs) should theoretically be different from that of tumor‐induced G‐MDSCs. Therefore, it is necessary to distinguish M‐MDSCs from G‐MDSCs when exploring the miRNAs that regulate their functions. In this study, we performed miRNA microarray analysis on both M‐MDSCs from tumor‐bearing mice (TM‐MDSCs) and their counterparts (CD11b^+^Ly6G^−^Ly6C^hi^ cells) from tumor‐free mice to identify differentially expressed miRNAs between TM‐MDSCs and their counterparts. Target genes of the identified miRNAs were predicted, among which genes that regulate proliferation and differentiation of myeloid cells were found by bioinformatics analyses. The functions of candidate target genes and associated miRNAs were further validated experimentally.

## 2 MATERIALS AND METHODS

### 2.1 Cell lines

Lewis lung carcinoma (LLC, Catalog number: TCM 7), B16 melanoma (Catalog number: TCM 2) and 293T cell lines (Catalog number: GNHu17) were purchased from cell resource center of Shanghai Institutes for Biological Sciences, Chinese Academy of Sciences. LLC and 293T cells were maintained in DMEM (Catalog number: SH30243.01B; HyClone, Logan City, Utah, USA), while B16 melanoma cells were cultured in RPMI 1640 (Catalog number: SH30809.01B; HyClone). Both culture mediums were supplemented with 10% fetal bovine serum (Catalog number: 10099‐141; GIBCO, Grand Island, New York, USA) and 1% penicillin‐streptomycin (Catalog number: 15140‐122; GIBCO).

### 2.2 Animals and establishment of tumor‐bearing mouse models

Female C57BL/6 mice, 4‐6 weeks of age, were purchased from the department of laboratory animal science of Fudan University (Shanghai, China) and were housed under specific pathogen‐free (SPF) conditions. Tumor‐bearing mouse models were created by injecting 1 × 10^6^ LLC cells or 1 × 10^5^ B16 cells subcutaneously into the flank. When tumor size reached 15‐20 mm in diameter, within 3‐4 weeks after injection, mice were euthanized with sodium pentobarbital. All animal experiments were conducted in accordance with the protocol for animal use and handling. This study was approved by the Animal Welfare and Ethics Committee of the Department of Laboratory Animal Science, Fudan University (permit no. 20150295A010).

### 2.3 Isolation of M‐MDSCs

To obtain individual splenic cells, murine spleens were mechanically dissociated, passed through a 75‐μm cellular sieve and then through a 30‐μm nylon mesh (Catalog number: 130‐041‐407; Pre‐Separation Filters; MiltenyiBiotec, Bergisch Gladbach, Germany). Red blood cells (RBC) were lysed with ACK Lysis Buffer (Catalog number: 420301; BioLegend, San Diego, California, USA). Cells were washed with phosphate buffered saline (PBS) and counted. Splenic cells were marked with specific fluorophore‐conjugated anti‐mouse antibodies in PBS for 15 minutes in the dark. Fluorophore‐conjugated antibodies used in this study include CD11b‐APC (Catalog number: 17‐0112‐81; M1/70; eBioscience, San Diego, California, USA), Ly6G‐PE (Catalog number: 551461; 1A8; BD Biosciences, Franklin Lakes, New Jersey, USA), and Ly6C‐PerCP‐Cy5.5 (Catalog number: 45‐5932‐82; HK1.4; eBioscience). M‐MDSCs were isolated by MoFloFlow Cytometry (Dakocytomation), and cell debris was excluded using a SSC/FSC gate. CD11b‐positive cells were gated, and then Ly6G^−^Ly6C^high^ cells were isolated as M‐MDSCs or their counterparts (from tumor‐free mice) and Ly6G^+^Ly6c^+/low^ cells as G‐MDSCs or their counterparts. Purity was over 90%.

### 2.4 RNA extraction and miRNA microarray assay

M‐MDSCs and their counterparts were stored in Trizol LS (Catalog number: 1596‐026; Invitrogen, Carlsbad, California, USA). Total RNA, including miRNAs, was isolated and purified using a miRNeasy Mini Kit (Catalog number: 217004; Qiagen, Hilden, Germany) and RNase‐free DNase I (Catalog number: 79254; Qiagen) following the manufacturer's instructions. Qualified RNA samples were measured using the miRNA array using GeneChip miRNA 3.0 Array (Catalog number: 902017; Affymetrix, Santa Clara, California, USA). Labeling and hybridization were conducted following the manufacturer's protocols. A GeneChip Scanner (GeneChip2 Scanner 30007G; Affymetrix) was used to scan signals on the slides. Feature extraction was performed using Affymetrix GeneChip Command Console software (AGCC). Raw data were processed using Expression Console Software (Affymetrix). First, raw data was normalized using a quantile algorithm. Probes with at least 100.0 percent of samples in any 1 condition out of 2 conditions that were flagged as “Detected” were chosen for further analysis. Differentially expressed miRNAs were then identified by fold change as well as *P*‐value calculation using the *t* test. Threshold for up‐ and down‐regulated genes was set as a fold change ≥2.0 and a *P* value ≤ 0.05. MicroRNA expression profiles of granulocytic MDSCs have been reported in our previous publication.^19^

### 2.5 Bioinformatics analysis

To identify miRNAs and their target genes that regulate proliferation and differentiation in M‐MDSCs, we predicted the target genes of differentially expressed miRNAs between TM‐MDSCs and their counterparts screened by microarray assay using miRwalk online software (http://www.umm.uni-heidelberg.de/apps/zmf/mirwalk). We also selected genes that regulate the proliferation and differentiation of myeloid cells using Ingenuity Pathway Analysis (IPA) online software (http://www.ingenuity.com/products/ipa). We integrated genes identified by both miRwalk and IPA software, and only overlapping genes were considered as candidates. Thus, corresponding miRNAs were considered candidate miRNAs that could regulate proliferation and differentiation of tumor‐induced M‐MDSCs and myeloid cells.

### 2.6 Real‐time quantitative PCR

Total RNA was isolated from cells using TRIzol^®^ (Catalog number: 1596‐026; Invitrogen) according to the manufacturer's protocol. RNA yield was determined using a NanoDrop 2000 spectrophotometer (Thermo Scientific, Waltham, Massachusetts, USA), and integrity was evaluated using agarose gel electrophoresis stained with ethidium bromide. Quantification was performed with a two‐step reaction process: reverse transcription (RT) and PCR. RT reactions were performed in a GeneAmp^®^ PCR System 9700 (Applied Biosystems, Foster City, California, USA) for 60 minutes at 37°C, followed by heat inactivation of RT for 5 minutes at 95°C. PCR reactions were incubated in a 384‐well optical plate (Roche, Basel, Swiss) at 95°C for 10 minutes, followed by 40 cycles of 95°C for 10 seconds, 60°C for 30 seconds. Complementary DNA was synthesized from 1 μg total RNA using a miScriptII Reverse Transcriptase Mix (Catalog number: 218161; Qiagen). Real‐time quantitative polymerase chain reaction (RT‐qPCR) was conducted using the LightCycler 480 SYBR Green I Master kit (Roche) protocol on a LightCycler 480 II RT‐PCR platform (Roche). Amplification of U6 small RNA (for mature miR‐486) and GAPDH mRNA (for CCAAT/enhancer‐binding protein‐alpha [*Cebpa*]) was performed with each experimental sample as an endogenous control. All reactions were run in triplicate. Relative expression levels were calculated using the comparative 2^−ΔΔCt^ method. Primer sequences were designed and synthesized by Generay Biotech (Generay, Shanghai, PRC) based on mRNA sequences obtained from the miRBase database (for miR‐486) and NCBI database (for *Cebpa*). The primer sequence of miR‐486 was 5′‐tcctgtactgagctgccccgag‐3′, and the reverse primer of miR‐486 was provided by Qiagen (Catalog number: 218161; Qiagen), which is proprietary. The forward primers for *Cebpa* and GAPDH were 5′‐tgagtgaggctctcattctt‐3′ and 5′‐atcactgccacccagaag‐3′, respectively. The reverse primers for *Cebpa* and GAPDH were 5′‐acatacacccttggacaacta‐3′ and 5′‐cagggatgatgttctgggca‐3′, respectively.

### 2.7 Construction of lentiviral vector

A genomic fragment of the mmu‐miR‐486 precursor from mouse chromosome was amplified. PCR primers were 5′‐tctagataactgagccaaggatgggtgggccag‐3′ and 5′‐gcctagggcggccgctcaggggtgggggtgggt‐3′. The PCR product was cloned into the pCDH vector (Catalog number: CD511B‐1; SBI, Mountain View, California, USA) by fusion cloning. For overexpression of *Cebpa*, a sequence encoding the CDS region of *Cebpa* was cloned into the GV287 vector (Catalog number: GV287; Shanghai Genechem Co., Ltd, Shanghai, China). PCR primers were 5′‐tggccccgtgaaaaatga‐3′ and 5′‐ggaggtgcaaaaagcaaggg‐3′. Then, the vectors and pPACK packaging plasmid mix (pCMV‐R8.92 and pVSVG‐I from Shanghai Holly Biotech Co., Ltd. Shanghai, China) were co‐transfected into 293T cells with Lipofectamine 2000 (Catalog number: 11668019; Invitrogen). Forty‐eight hours later, viral particles were collected from the supernatant and subsequently purified. After titer determination, virus was stored in single use aliquots for future use at −80°C to reduce viral titer loss from freeze‐thaw cycles.

### 2.8 Construction of *Cebpa* 3′‐UTR luciferase reporter vector pGL‐*Cebpa*‐3′UTR

As revealed by software analysis, the mmu‐miR‐486‐5p has one potential binding site in the 3′‐UTR of *Cebpa*. The wild type 3′‐UTR fragment of *Cebpa* was amplified from mouse genomic DNA using PrimeSTAR^®^ HS DNA Polymerase (Catalog number: R010A; TakaRa, Tokyo, Japanese) and was purified from agarose gels using TIANgel Midi Purification Kit (Catalog number: DP‐209; TakaRa, Japanese). Primer sequences are as follows: 5′‐gatcgccgtgtaattctagaggcgcgcggctgcggg‐3′ and 5′‐gccggccgccccgacttgagtttgatatgtttatattat‐3′. Next, mutation was introduced to the potential mmu‐miR‐486‐5p binding site in the 3′‐UTR of *Cebpa*. The mutant miR‐486‐5p binding site was created using the following primers: 5′‐gatctcagcttcgacattcttcctcct‐3′ and 5′‐aggaggaagaatgtcgaagctgagatc‐3′. The pGL‐3‐promoter was linearized with endonuclease XbaI (Catalog number: R0145L; NEB, Ipswich, Massachusetts, USA). All amplified fragments were inserted into the pGL‐3‐promoter (Catalog number: E1761; Promega, Madison, Wisconsin, USA), yielding vectors pGL‐*Cebpa*‐3′UTR‐WT and pGL‐*Cebpa*‐3′UTR‐MT (WT: wild type, MT: mutant). All amplified fragments were inserted into the pGL‐3‐promoter by infusion cloning using the NovoRec recombinase polymerase kit (Catalog number: NR001B; Sinobio, Hong Kong, China) and transformed into DH5 α competent cells. Positive clones were identified by PCR, and sequences were analyzed. Lentivirus plasmids containing target genes were constructed.

### 2.9 Luciferase reporter assays

293T cells were plated into 24‐well plates at a density of 30%‐50% 1 day before transfection. 293T cells were then cotransfected with pGL‐*Cebpa*‐3′UTR‐WT or pGL‐*Cebpa*‐3′UTR‐MT, mmu‐miR‐486 mimic (Catalog number: miR10003130‐1‐5; Guangzhou RiboBio, Guangzhou, China) or mimic NC (negative control; Catalog number: 804005; Shanghai GenePharma Co., Ltd, Suzhou, China), and reporter plasmid pRL‐TK (Catalog number: E2231; Promega) with renilla luciferase using Lipofectamine 2000 reagent (Catalog number: 11668019; Invitrogen) according to the manufacturer's protocol. Twenty‐four hours after transfection, both renilla and firefly luciferase activities were detected using a dual luciferase kit (Catalog number: E1910; Promega). Renilla luciferase activity was normalized based on the activity of firefly luciferase.

### 2.10 Isolation and culture of myeloid cells

C57BL/6 mice were anesthetized with 2% pentobarbital sodium and sterilized using 75% ethylalcohol for 10 minutes. Both femora and tibias were isolated under aseptic conditions, and the epiphyses were subsequently removed. Bone marrow was washed away using RPMI‐1640 (Catalog number: SH30809.01B; HyClone) with 10% fetal bovine serum (Catalog number: 10099‐141; GIBCO) and 1% penicillin‐streptomycin (Catalog number: 15140‐122; GIBCO). Bone marrow was triturated and RBCs were lysed with ACK Lysis Buffer (Catalog number: 420301; BioLegend). Bone marrow cells were cultured overnight in RPMI‐1640 with 10% fetal bovine serum and 1% penicillin‐streptomycin. Then, both 40 ng/mL GM‐CSF (Catalog number: 83869‐56‐1; PEROTECH, USA) and 40 ng/mL IL‐6 (Catalog number: 216‐16; Princeton, New Jersey, PEROTECH) were added for 4 days to induce myeloid cell differentiation into MDSCs.^13^

### 2.11 Transfection of myeloid cells with lentivirus

Total nucleated myeloid cells, that had been treated with GM‐CSF and IL‐6 for 4 days, were cultured in 6‐well plates at 3 × 10^5^/mL per well for 24 hours and transfected the following day without GM‐CSF and IL‐6. Lentivectors were added to infect myeloid cells for 48 hours and these cells were subsequently analyzed for proliferation, differentiation and apoptosis.

### 2.12 Flow cytometry analysis of cell proliferation, apoptosis, and cell surface proteins

Flow cytometry was performed using a BD flow cytometer (Accuri C6). Antibodies used for FACS staining were anti‐mouse CD11b‐APC (Catalog number: 17‐0112‐81; eBioscience), Ly6G‐PE (Catalog number: 551461; BD Biosciences), Ly6C‐PerCP‐Cy5.5 (Catalog number: 45‐5932‐82; eBioscience), CD11c‐FITC (Catalog number: 130‐102‐466; MiltenyiBiotec), F4/80‐FITC (Catalog number: 130‐102‐988; MiltenyiBiotec), CD80‐FITC (Catalog number: 11‐0801‐81; eBioscience), CD86‐FITC (Catalog number:11‐0862‐81; eBioscience). BrdU Staining Kit‐FITC (Catalog number: 8811‐6600; eBioscience) and Annexin V‐FITC Apoptosis Detection Kit (Catalog number: C1062; Beyotime Biotechnology, Shanghai, China) were used to measure cell proliferation and apoptosis, respectively. After intervention and subsequent culturing, all nucleated myeloid cells were separated into suspended cells and adherent cells. Suspended cells were stained with CD11c‐FITC, CD80‐FITC, and CD86‐FITC, and adherent cells were stained with F4/80‐FITC. All nucleated myeloid cells, including both suspended cells and adherent cells, were used to measure expression of CD11b‐APC, Ly6G‐PE, and Ly6C‐PerCP‐Cy5.5, cell proliferation and apoptosis. The results are represented as percentages relative to the total number of positive cell proportions of all cells analyzed by FACS. For FACS, cell suspensions were centrifuged, and the supernatant was aspirated off completely. Cells were resuspended to 10^5‐6^ cells in a final volume of 100 μL PBS. Fluorophore‐conjugated anti‐mouse antibodies were added. The final dilution ratios of fluorophore‐conjugated antibodies by PBS were 200, 200, 20, 10, 10, 200, 400, 20, 5, and 20 for CD11b, Ly6G, Ly6c, CD11c, F4/80, CD80, CD86, BrdU, Annexin V and PI, respectively. Then, cells were incubated for 15 minutes in the dark in the refrigerator (2‐8°C). Cells were washed once before detection, cells debris was excluded using a SSC/FSC gate. Representative gating of FACS plots for BrdU, Annexin V, PI, CD11c, CD80, CD86, F4/80, CD11b, Ly6G, and Ly6c are shown in Figure S2.

### 2.13 Western blotting analysis

Cells were lysed using RIPA lysis buffer (Catalog number: BYL40825; JRDUN Biotech, Shanghai, China) containing both protease and phosphatase inhibitors. Cell lysates for western blot analysis were denatured and subjected to SDS‐PAGE. According to the molecular weight of Cebpa, which is 43 kDa, we chose a 10% gel. The semi‐dry method was used to transfer membranes, and 30 μg per well of protein was utilized. Proteins were transferred to nitrocellulose filter membranes (Catalog number: HATF00010; Millipore, USA), and membranes were blocked with 5% skim milk powder overnight at 4°C. Membranes were then incubated with primary antibodies against *Cebpa* (Catalog number: ab40764; Abcam, Cambridge, UK) and GAPDH (Catalog number: 5174; CST, Boston, Massachusetts, USA), which were diluted 500 times and 1500 times, respectively, in PBS containing 5% skim milk powder, according to their manual instructions and were incubated with membranes at room temperature for 2 hours. Membranes were then hybridized with secondary HRP‐conjugated antibodies (Catalog number: A0208; Beyotime Biotechnology), which were diluted 1000 times according to manual instruction in TBST at 37°C for 1 hour. Protein‐Ab complexes were detected using enhanced chemiluminescence (ECL; Catalog number: WBKLS0100; Millipore). The luminescence signal was visualized on a chemiluminescent imaging system (Model: Tanon 5200; Tanon Science & Technology Company Limited, Shanghai, China).

### 2.14 Statistical analysis

Results are presented as the mean ± SD. Data were analyzed using GraphPad Prism 6 software for Windows (GraphPad Software, Inc, La Jolla, CA, USA). Statistical analyses were performed using Stata/SE 10.1 software (Statacorp, College Station, Texas, USA). A two‐tailed Student's *t* test was used to compare data between two groups, and one‐way ANOVA was used to compare data between three groups with the Scheff multiple‐comparison test. For miRNA microarray assay, fold changes of 2 or more and a *P*‐value < 0.05 were considered statistically significant. For all other tests, *P* < 0.05 was considered statistically significant.

## 3 RESULTS

### 3.1 Differentially expressed miRNAs between TM‐MDSCs and their counterparts

**Figure 1:**
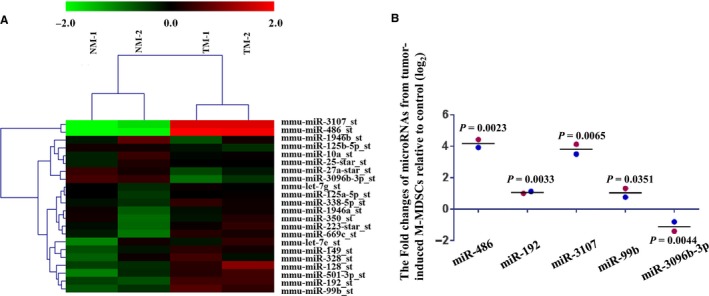
Differentially expressed miRNAs between TM‐MDSCs their counterparts. A, M‐MDSCs from spleens of LLC‐bearing mice (TM) and their counterparts from tumor‐free mice (NM) were purified using flow cytometry. MiRNA expression profiles were detected using Affymetrix GeneChip miRNA 3.0 Array. Results of the two‐way hierarchical clustering of genes and samples are displayed by heat map diagram, which was generated using the MutiExperiment Viewer (MEV) software. MiRNAs with fold changes ≥2.0 and *P*‐values < 0.05 were considered differentially expressed. The color scale at the top displays the relative miRNA expression level. Differential expression of up‐ (red) and down‐regulated (green) miRNAs between TM (TM‐MDSCs) and NM (their counterparts) groups are scaled according to the color code depicted. Each sample column represents data from total RNA purified from one independent cell sort using three to five mice. Five differentially expressed miRNAs were identified. MiR‐486, miR‐3107, miR‐192 and miR‐99b were expressed at higher levels in TM‐MDSCs, and miR‐3096b was expressed at a lower level in TM‐MDSCs compared with their counterparts (experiments were repeated twice). Other miRNAs included in the heat map diagram were overexpressed in tumor‐induced G‐MDSCs compared with their counterparts from tumor‐free mice. MicroRNA expression profiles of granulocytic MDSCs have been reported in our previous publication (See reference 19). B, The five differentially expressed miRNAs detected by microarray displayed by scatter plot. Horizontal lines signify mean value, and red plot and blue plot indicate different biological replicates

M‐MDSCs from the spleens of LLC‐bearing mice (TM‐MDSCs) and their counterparts (CD11b^+^Ly6G^−^Ly6c^hi^ cells from spleens of tumor‐free mice) were purified using flow cytometry. The gating strategy used to isolate M‐MDSCs is shown in Figure S1 and in our previous paper.^19^ MiRNA expression profiles were generated using Affymetrix GeneChip miRNA 3.0 Array. The results of the two‐way hierarchical clustering of genes and samples are displayed as a heat map diagram (Figure 1A), which was generated with the MutiExperiment Viewer (MEV) software. Five differentially expressed miRNAs were detected between TM‐MDSCs and their counterparts in the microarray assay. MiR‐486, miR‐3107, miR‐192, and miR‐99b were expressed at higher levels in TM‐MDSCs, and miR‐3096b was expressed at a lower level in TM‐MDSCs than their counterparts. Figure 1A also shows another 17 microRNAs that were not differentially expressed between TM‐MDSCs and their counterparts but that were overexpressed in tumor‐induced G‐MDSCs compared with their counterparts (CD11b^+^Ly6G^+^Ly6c^low/+^ cells) from tumor‐free mice (Fold changes ≥ 2.0 and *P*‐value < 0.05 found by microarray).^19^ The five differentially expressed miRNAs between TM‐MDSCs and their counterparts as detected by microarray are also displayed by scatter plot (Figure 1B). To explore these miRNAs and their target genes that regulate the proliferation and differentiation of TM‐MDSCs, we predicted target genes for the differentially expressed miRNAs between TM‐MDSCs and their counterparts using miRwalk online software. Since MDSCs are derived from myeloid cells, we picked genes that regulate the proliferation (108 genes) and differentiation (346 genes) of myeloid cells using IPA online software. We integrated genes identified by both miRwalk and IPA software, and only overlapping genes were considered candidates. *Cebpa* was predicted to be the only gene that could regulate the proliferation of TM‐MDSCs and myeloid cells, and the corresponding miRNA was miR‐486.

### 3.2 Overexpression of miR‐486 promotes proliferation, and suppresses apoptosis and differentiation in myeloid cells

**Figure 2:**
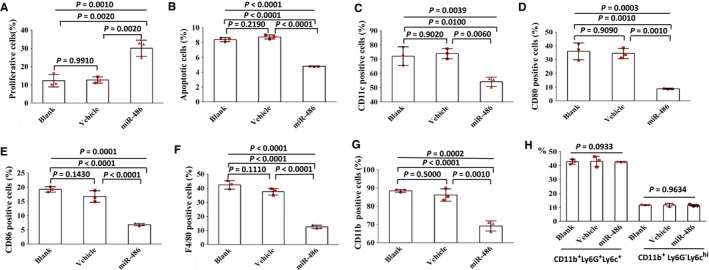
Overexpression of miR‐486 promotes proliferation and suppresses apoptosis and differentiation in myeloid cells. Murine myeloid cells were transfected with vehicle lentivirus or miR‐486‐expressing lentivirus for 48 h, or left without treatment (blank control), and then BrdU, Annexin V, or associated antibodies were added. Cells were tested by flow cytometry. A. Results show that miR‐486 promotes proliferation of myeloid cells. B. Results show that miR‐486 suppresses apoptosis of myeloid cells. C‐H. Expression of CD11c, CD80, CD86, F4/80, and CD11b were suppressed by miR‐486. Overexpression of miR‐486 did not change the percentage of CD11b^+^Ly6G^+^Ly6C^low^ G‐MDSCs and CD11b^+^Ly6G^−^Ly6C^hi^ M‐MDSCs. All data are presented as the mean ± SD and were repeated three times, and error bars represent SD. Each red scatter plot overlaid onto the solid bar graphs indicates one technical repeat. The *P*‐values above the horizontal lines without vertical bars represent the results of one‐way ANOVA for three groups, and the other *P*‐values represent results of the Scheff multiple‐comparison test when *P*‐values were lower than 0.05 in the one‐way ANOVA analyses

Myeloid cells from mice, that included all myeloid cells, with the exception of red blood cells, were transfected with vehicle lentivirus or miR‐486‐expressing lentivirus for 48 hours, or were left without treatment (blank control). Cells were examined by flow cytometry. The results showed that overexpression of miR‐486 promoted the proliferation of myeloid cells (Figure 2A) and suppressed the apoptosis of myeloid cells (Figure 2B). The expressions levels of CD11c, CD80, CD86, F4/80, and CD11b were suppressed by miR‐486. Both CD11b^+^Ly6G^+^Ly6C^low^ cells (phenotype of G‐MDSCs) and CD11b^+^Ly6G^−^Ly6C^hi^ cells (phenotype of M‐MDSCs) were comparable between the three groups. The data indicate that overexpression of miR‐486 inhibits myeloid differentiation (Figure 2C‐H).

### 3.3 Expression of miR‐486 is inversely correlated with the potential target gene *Cebpa* in M‐MDSCs

**Figure 3:**
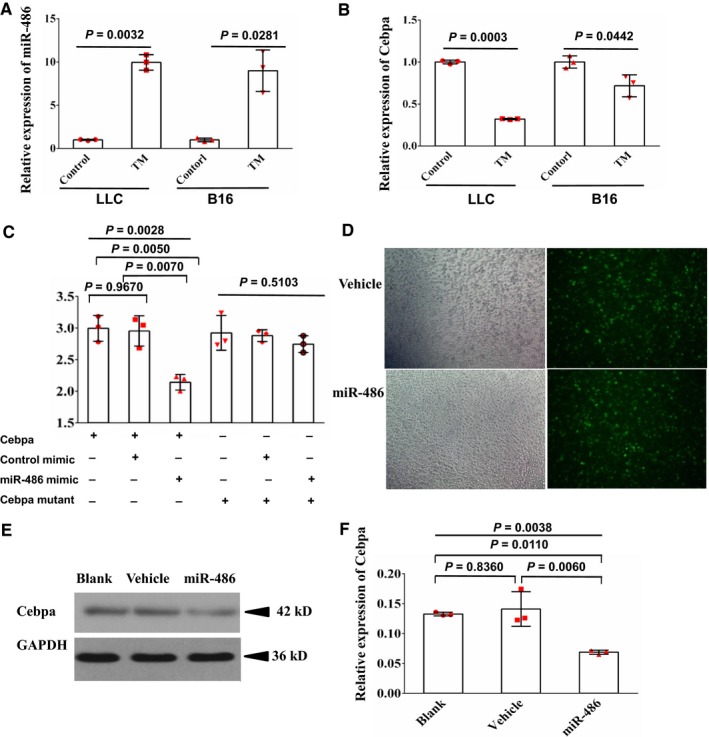
MiR‐486 inhibits expression of *Cebpa*. A, Expression of miR‐486 in TM‐MDSCs from spleens of both LLC‐bearing mice and B16 melanoma‐bearing mice was higher than in control groups (their counterparts from spleens of tumor‐free mice) as determined by RT‐qPCR. B, Expression of *Cebpa*, which was predicted as one target of miR‐486 by bioinformatics, was lower in TM‐MDSCs from both LLC‐bearing mice and B16 melanoma‐bearing mice than in control groups. C, Interaction between miR‐486 and *Cebpa* was detected using the Promega luciferase reporter assay, showing that the miR‐486 mimic, but not control miRNA, significantly reduced renilla luciferase activity in wide type 3′‐UTR *Cebpa* groups; however, neither the miR‐486 mimic, nor control miRNA, significantly reduced renilla luciferase activity in mutated 3′‐UTR Cebpa groups. D, Transfection efficiency in murine myeloid cells infected with vehicle lentivirus or miR‐486‐expressing lentivirus observed by a fluorescence microscope. Original magnification ×100. E, Murine myeloid cells were infected with vehicle lentivirus or miR‐486‐expressing lentivirus for 48 h. Expression of *Cebpa* in these cells was measured by western blot. The results also indicated that miR‐486 reduced the expression of *Cebpa* in myeloid cells. F, Overexpression of miR‐486 decreased the expression of *Cebpa* in myeloid cells as shown by RT‐qPCR. Three tumor‐bearing mice and five tumor‐free mice were used for each test in A and B. All data are presented as the mean ± SD and repeated three times. Error bars represent SD. Each red scatter plot overlaid onto the solid bar graphs indicates one technical repeat. For A and B, a two‐tailed Student's *t* test was used to compare data between two groups. For C and F, the *P*‐values above the horizontal lines without vertical bars represent the results of one‐way ANOVA for three groups, and other *P*‐values represent the results of the Scheff multiple‐comparison test when the *P*‐values were lower than 0.05 in the one‐way ANOVA analyses

To explore the relationship between miR‐486 and *Cebpa* in M‐MDSCs, we measured miR‐486 and *Cebpa* expression in TM‐MDSCs from both LLC‐bearing mice and B16‐bearing mice. The results showed that the expression of miR‐486 in TM‐MDSCs from both LLC‐bearing mice and B16 melanoma‐bearing mice was higher than that in their counterparts (Figure 3A), which also validated our microarray data. Additionally, the expression of *Cebpa* was lower in TM‐MDSCs from both LLC‐bearing mice and B16 melanoma‐bearing mice than in their counterparts (Figure 3B).

### 3.4 Overexpression of miR‐486 inhibits expression of *Cebpa*

To assess whether miR‐486 directly regulates the expression of *Cebpa*, a fragment of the 3′‐UTR of *Cebpa* mRNA, containing the putative miR‐486‐binding sequence (wild type) or a mutated binding site (mutant), was cloned into a luciferase reporter construct and cotransfected with a control renilla luciferase reporter plasmid into 293T cells along with control or miR‐486 mimic. Renilla luciferase activity served as an internal control. We found that the miR‐486 mimic, but not the control miRNA, significantly reduced renilla luciferase activity in the wild‐type 3′‐UTR *Cebpa* groups; however, neither the miR‐486 mimic nor the control miRNA significantly reduced renilla luciferase activity in the mutant 3′‐UTR *Cebpa* groups, suggesting that miR‐486 regulates gene expression directly at the *Cebpa* 3′‐UTR (Figure 3C). We next investigated the role of miR‐486 in the regulation of *Cebpa* in myeloid cells. Murine myeloid cells were isolated and cultured in RPMI‐1640, and then, the cells were infected with miR‐486‐expressing lentivector, vehicle lentivirus, or left without treatment (blank) for 48 hours. The transfection efficiency of the murine myeloid cells infected with vehicle lentivirus or miR‐486‐expressing lentivirus was observed by fluorescence microscopy (Figure 3D). The expression of *Cebpa* in these cells was measured by both Western‐blot and RT‐qPCR. The results indicated that the overexpression of miR‐486 inhibits the expression of *Cebpa* in myeloid cells (Figure 3E,F). Original uncropped and unprocessed western blot images are provided in Figure S3.

### 3.5 Overexpression of *Cebpa* induces apoptosis and suppresses proliferation and differentiation of myeloid cells

**Figure 4:**
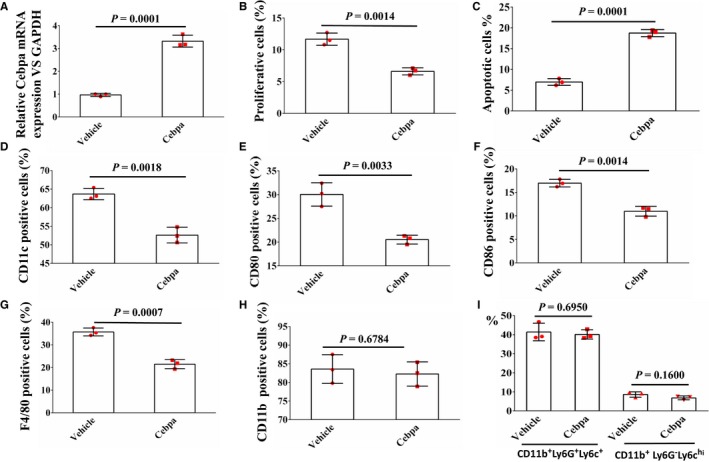
Overexpression of *Cebpa* induces apoptosis and suppresses proliferation and differentiation in myeloid cells. Murine myeloid cells were transfected with vehicle lentivirus or *Cebpa*‐expressing lentivirus for 48 h. A, Overexpression of *Cebpa* measured by RT‐qPCR. B, Overexpression of *Cebpa* suppresses proliferation of myeloid cells. C. Overexpression of *Cebpa* promotes apoptosis of myeloid cells. D‐I, Overexpression of *Cebpa* reduces the expression of CD11c, CD80, CD86 and F4/80 but has no effect on the proportions of both G‐MDSCs and M‐MDSCs compared with the vehicle group. All data are presented as the mean ± SD and were repeated three times. Error bars represent SD. Each red scatter plot overlaid onto the solid bar graphs indicates one technical repeat. A two‐tailed Student's *t* test was used to compare data between two groups

Murine myeloid cells were transfected with vehicle lentivirus or *Cebpa*‐expressing lentivirus for 48 hours, and overexpression of *Cebpa* was subsequently confirmed by RT‐qPCR (Figure 4A). Overexpression of *Cebpa* suppressed proliferation (Figure 4B) and promoted apoptosis in myeloid cells (Figure 4C). Furthermore, overexpression of *Cebpa* reduced the expression levels of CD11c, CD80, CD86, and F4/80 but had no effect on the percentage of CD11b^+^Ly6G^+^Ly6C^low^ cells and CD11b^+^Ly6G^−^Ly6C^hi^ cells compared with the vehicle group, indicating that overexpression of *Cebpa* inhibits myeloid cell differentiation (Figure 4D‐I).

## 4 DISCUSSION

MDSCs are a heterogeneous population of activated immature myeloid cells that are characterized by a morphological mixture of granulocytic and monocytic cells but lack expression of cell‐surface markers associated with fully differentiated monocytes, macrophages or dendritic cells. MDSCs can be categorized into two major groups, namely G‐MDSCs and M‐MDSCs, based on their cell‐surface markers expression profiles.^7^ Increasing evidence indicates that MDSCs play important roles in the progression of cancer in both tumor‐bearing mice and humans. Apart from their powerful potential to suppress immune responses, recent studies have suggested that MDSCs might be directly incorporated into the tumor endothelium, secreting many pro‐angiogenic factors, inducing the production of matrix metalloproteinases (MMPs) and chemoattractants, and establishing a premetastatic environment.^20^ Since they play crucial roles in tumor immunotolerance, angiogenesis and tumor metastasis, MDSCs are considered a promising antitumor target. Several hypotheses have been developed for targeting MDSCs, including promoting the differentiation of MDSCs into mature and non‐suppressive cells, decreasing the number of MDSCs and functionally inhibiting MDSCs.^21^

Since two main characteristics of MDSCs are marked accumulation and immaturity, understanding the mechanisms that regulate the proliferation and differentiation of MDSCs is important for targeting these cells. Preliminary data from previous studies has shown that miRNAs seem to have the potential to increase MDSC accumulation in both human cancers and tumor‐bearing mice.^7^, ^13^ As M‐MDSCs have some biological characteristics that are different from those of G‐MDSCs, the miRNAs that regulate proliferation and differentiation of M‐MDSCs may be different from those of G‐MDSCs. To screen candidate miRNAs that regulate the proliferation and differentiation of TM‐MDSCs, we performed miRNA microarray analysis to identify differentially expressed miRNAs between TM‐MDSCs and their counterparts (CD11b^+^Ly6G^−^Ly6c^hi^ cells from tumor‐free mice). Five miRNAs were identified, and of these, miR‐486 was overexpressed in TM‐MDSCs compared with their counterparts. MicroRNA expression profiles of G‐MDSCs compared with their counterparts from tumor‐free mice have been reported in our previous publication.^19^ According to the criteria that fold changes should be no less than 2, we found that G‐MDSCs overexpressed 20 microRNAs and that no microRNAs were lower in expression levels than their counterparts. Among the 20 overexpressed microRNAs in G‐MDSCs, miR‐486, miR3107 and miR‐192 were also overexpressed in M‐MDSCs. However, overexpression of miR‐486 in G‐MDSCs was not validated using RT‐PCR.^19^ The differential microRNA expression profiles between G‐MDSCs and M‐MDSCs indicated that G‐MDSCs and M‐MDSCs share some common microRNAs while also exhibiting distinct microRNAs.

Unfortunately, obtaining enough M‐MDSCs for culture in vitro is technically challenging. Furthermore, maintaining the properties and activity of M‐MDSCs and infecting using lentivirus in vitro is also difficult. As M‐MDSCs are derived from myeloid cells, we studied the role of miR‐486 in myeloid cells and indirectly speculated the roles of miR‐486 and Cebpa in M‐MDSCs. In our study, we used GM‐CSF/IL‐6 to induced myeloid cell differentiation into MDSCs in vitro, which was reported by Li and his colleagues.^13^ In their study, they found that this culture could induce expression of miR‐155 and miR‐21 as well as the immunosuppression function of MDSCs, which mirrors the profile of MDSCs in vivo induced by tumor.^13^ In their study, they did not observe high expression of miR‐486 in these cultured cells, which included G‐MDSCs and M‐MDSCs, using microarrays, in contrast to our results in M‐MDSCs from the spleens of LLC‐bearing mice.^13^ In this study, we found that overexpression of miR‐486 in myeloid cells promoted proliferation and suppressed apoptosis and differentiation of these cells.

The target genes of miR‐486 that might regulate the proliferation and differentiation of myeloid cells were predicted by bioinformatics analyses. *Cebpa* was predicted to be a major target gene that could regulate the proliferation of TM‐MDSCs. In our study, the expression of *Cebpa* was inversely correlated with miR‐486 in TM‐MDSCs measured by RT‐qPCR, indicating that TM‐MDSCs express a lower level of *Cebpa*. Hegde, and his colleagues also found that both M‐MDSCs (CD11b^+^Ly6G^−^Ly6C^+^) and G‐MDSCs (CD11b^+^Ly6G^+^Ly6C^+^) induced by 9‐Tetrahydrocannabinol (the major bioactive component of marijuana) from peritoneal exudates of mice expressed lower levels of Cebpa than did bone marrow precursors and that Cebpa might be regulated by miR‐690.^22^ In our study, the results from both luciferase reporter assays in 293T cells and overexpression of miR‐486 in myeloid cells indicated that miR‐486 inhibits expression of *Cebpa,* implying that *Cebpa* is a direct target of miR‐486.

A previous study demonstrated that the *Cebpa* gene is located on chromosome 19q13.1, belonging to the CCAAT/enhancer‐binding protein family, which act as a group of transcription factors with a pivotal role in the differentiation and proliferation of various cell types, including hematopoietic cells.^23^ Human *Cebpa* mRNA exists in myeloid cells but not in lymphoid cells. The expression of the human *Cebpa* gene starts at the time of stem cell differentiation into the myeloid lineage.^23^ Human *Cebpa* mRNA and protein were found to increase with granulocytic differentiation and to decrease with monocytic differentiation.^24^

Overexpression of *Cebpa* in myeloid cells suppresses proliferation and differentiation and promotees the apoptosis of these cells. The functions of *Cebpa* in suppressing cell proliferation and promoting apoptosis found in our studies were in accordance with published data. Down‐regulating *Cebpa* by CUGBP1 was reported to promote cell proliferation and suppress apoptosis in human non‐small cell lung cancers.^25^ Downregulation of *Cebpa* by IL‐17 promotes keratinocyte proliferation.^26^ It was also reported that the transcription factor *Cebpa* is a strong inhibitor of cell proliferation through cyclin‐dependent protein kinase 2 (cdk2) and cdk4.^27^ Overexpression of *Cebpa* significantly decreased cell proliferation and upregulated the expression levels of the apoptosis‐related genes Foxo3 and Bim in K562 cells.^28^ *Cebpa* siRNA was reported to upregulate iNOS and suppress apoptosis of hepatocytes.^29^ Previous research indicated that conditional expression of *Cebpa* alone in stably transfected bipotential (granulocytic/monocytic) myeloid cell line triggered neutrophilic differentiation, while blocking monocytic differentiation.^17^ In our study, overexpression of *Cebpa* suppressed the differentiation of myeloid cells but had no effect on the proportion of G‐MDSCs and M‐MDSCs. It was reported that *Cebpa* and Cebpb play pivotal and partially overlapping roles in determining airway epithelial differentiation.^30^ *Cebpa* suppresses macrophage differentiation by activating QKI‐5 and then down‐regulating CSF1R expression.^31^ Recently, Mackert and his colleagues also discovered that *Cebpa* expression was significantly reduced in MDSCs from tumor‐bearing mice compared to tumor‐free animals. Tumor‐conditioned medium could down‐regulated *Cebpa* expression. Myeloid lineage specific deletion of *Cebpa* resulted in significantly enhanced MDSC proliferation and expansion, as well as an increase in myeloid progenitors and a decrease in mature cells. In addition, tumors from *Cebpa* myeloid conditional null mice have increased MDSC infiltration and angiogenesis in vivo.^32^ Findings from Mackert and colleagues were coincident with ours. They also found that down regulation of Cebpa in myeloid cells blocks differentiation, which consistent with our findings that overexpression of miR‐486 suppresses the differentiation of myeloid cells by decreasing the expression of *Cebpa*. However, when we overexpressed *Cebpa* in myeloid cells, the differentiation of myeloid cells was still suppressed.^31^ In addition, Mackert and colleagues found that deletion of *Cebpa* in MDSCs enhanced the pro‐angiogenic, immunosuppressive and pro‐tumorigenic behavior of these cells by upregulating the production of iNOS and arginase, as well as MMP‐9 and VEGF.^32^

Our data show that suppressing the expression of *Cebpa* by miR‐486 and overexpressing of *Cebpa* inhibits the differentiation of myeloid cells, which seems inconsistent and demonstrates that these regulatory networks are complicated. Furthermore, there may be other target genes whose relationship with miR‐486 might be more crucial to cell differentiation, or *Cebpa* may exert bidirectional regulatory effects on the differentiation of myeloid cells. That is, both overexpression and depletion of *Cebpa* suppress the differentiation of myeloid cells. The mechanism of miR‐486‐ and *Cebpa‐*induced suppression of myeloid cell differentiation needs further study.

## 5 CONCLUSION

Our study indicates that miR‐486 promotes proliferation and suppresses apoptosis of myeloid cells by targeting *Cebpa*, which suppresses differentiation by other unknown target genes that need to be further elucidated. Additionally, miR‐486 and *Cebpa* might be involved in TM‐MDSC expansion in tumor‐bearing mice.

## CONFLICT OF INTEREST

None declared.
