# Supplementary figures and images for: MiR‐486 promotes proliferation and suppresses apoptosis in myeloid cells by targeting Cebpa in vitro
Source: Cancer Med. 2018 Aug 2;7(9):4627–38. doi: 10.1002/cam4.1694 (PMC6143942; doi:10.1002/cam4.1694)

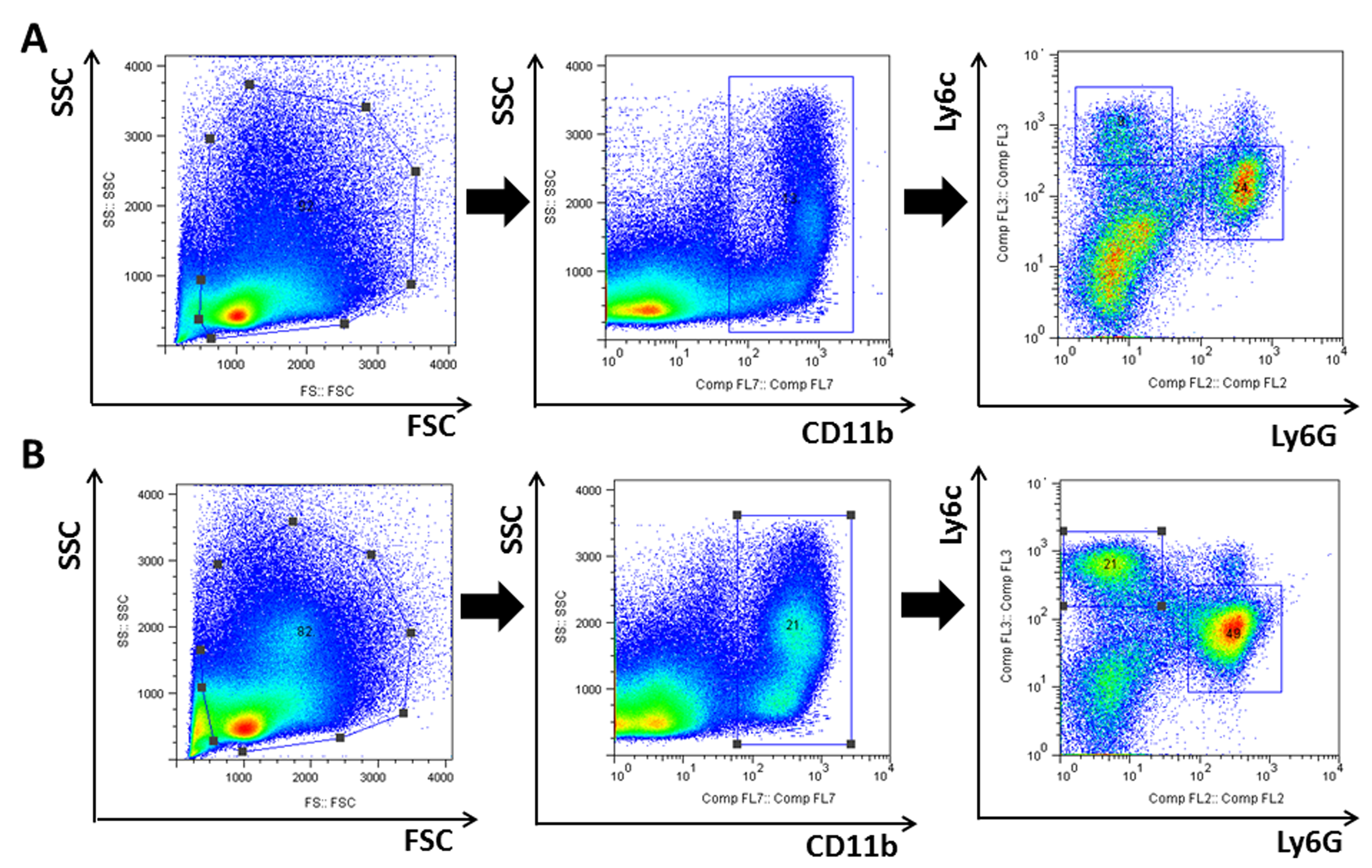

Supplement: Supplementary file 1 [file CAM4-7-4627-s001.tif]

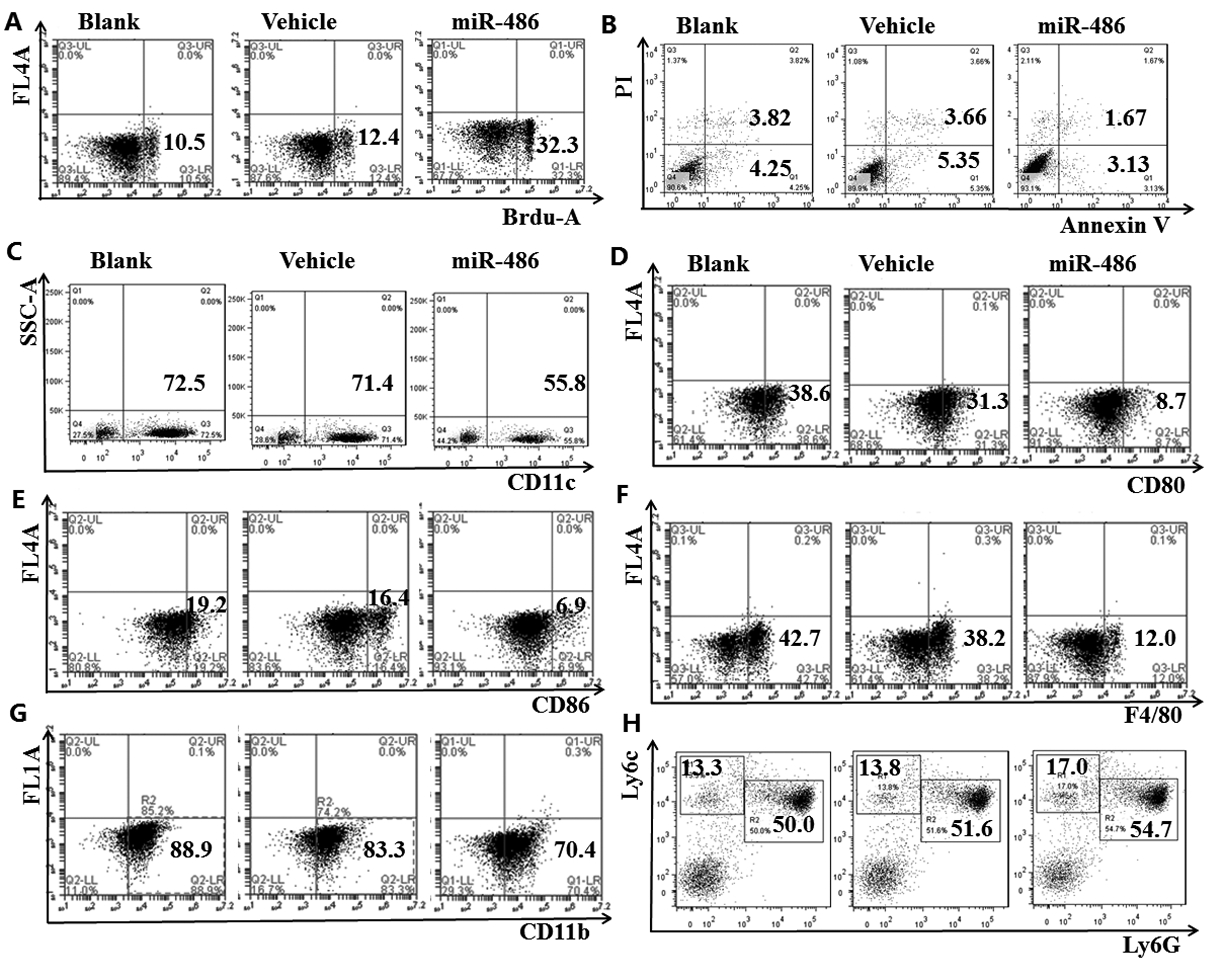

Supplement: Supplementary file 2 [file CAM4-7-4627-s002.tif]

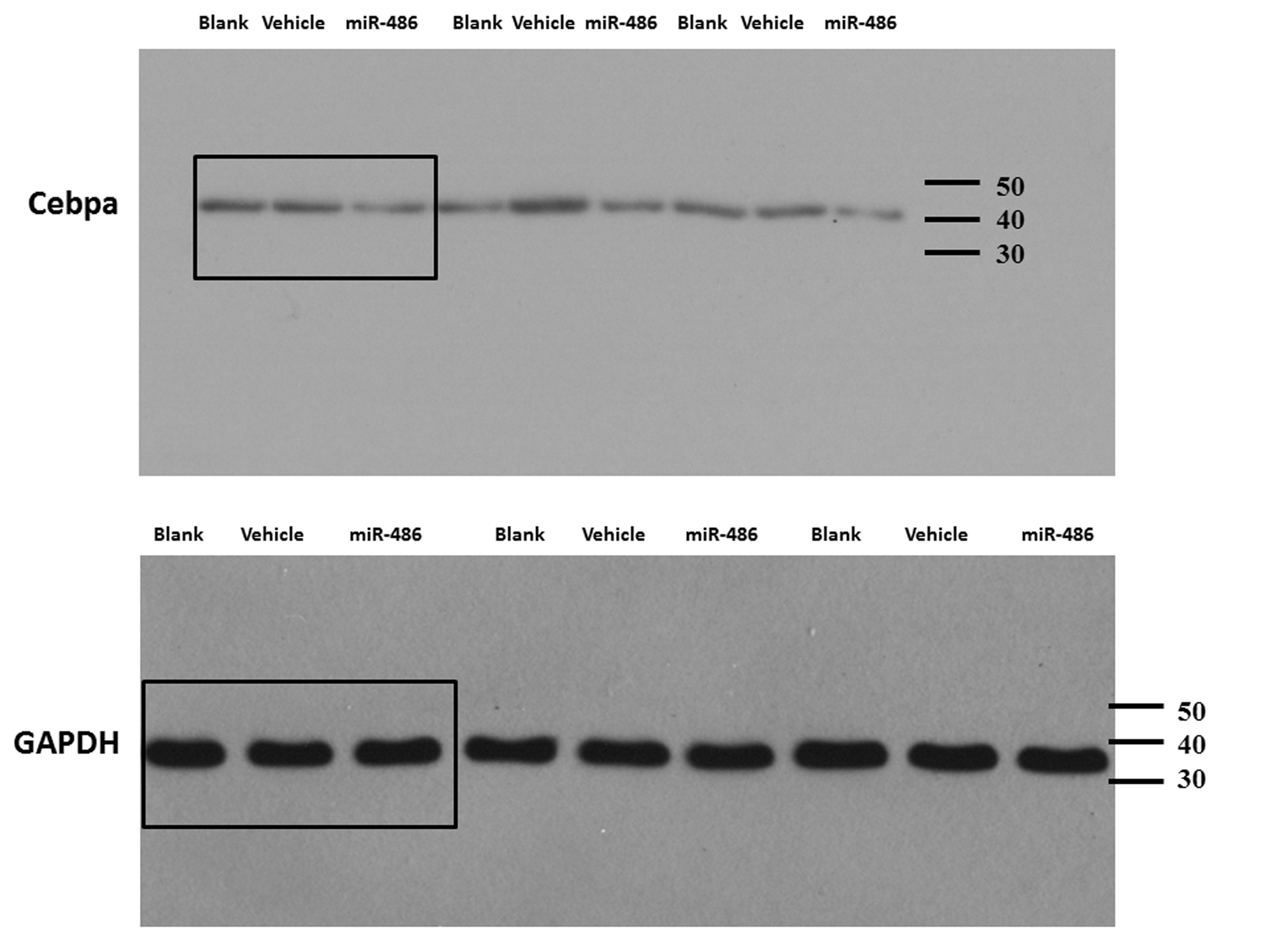

Supplement: Supplementary file 3 [file CAM4-7-4627-s003.TIF]
